# The transmission of visceral leishmaniasis in the municipality of Guarujá, on the Coast of São Paulo state, Brazil

**DOI:** 10.11606/s1518-8787.2022056003680

**Published:** 2022-02-09

**Authors:** Claudio Casanova, Gabriela Motoie, Maria de Fátima Domingos, Vanessa Gusmon da Silva, Mariana Dantas da Silva, Eunice Aparecida Bianchi Galati, Fredy Galvis-Ovallos

**Affiliations:** I Superintendência de Controle de Endemias São Paulo SP Brasil Superintendência de Controle de Endemias. São Paulo, SP, Brasil; II Instituto Adolfo Lutz São Paulo SP Brasil Instituto Adolfo Lutz. São Paulo, SP, Brasil; III Universidade de São Paulo Faculdade de Saúde Pública Programa de Pós-Graduação em Saúde Pública São Paulo SP Brasil Universidade de São Paulo. Faculdade de Saúde Pública. Programa de Pós-Graduação em Saúde Pública. São Paulo, SP, Brasil; IV Universidade de São Paulo Faculdade de Saúde Pública Departamento de Epidemiologia São Paulo SP Brasil Universidade de São Paulo. Faculdade de Saúde Pública. Departamento de Epidemiologia. São Paulo, SP, Brasil

**Keywords:** Leishmaniasis, Visceral, transmission, Disease Vectors, Psychodidae, parasitology, Disease Outbreaks

## Abstract

**OBJECTIVE:**

To perform an entomological survey, evaluating the circulation of *Leishmania* spp. in sand flies captured from the new foci of visceral leishmaniasis (VL) in the coastal region of São Paulo state.

**METHODS:**

Sand flies were captured from November 2016 to September 2018 using light traps of the Centers for Disease Control (CDC), in the neighborhood where VL cases were reported. *Leishmania* spp. circulation was evaluated by gut dissection and molecular analysis of the females captured.

**RESULTS:**

*Nyssomyia intermedia* was the more frequent species (90,7%) within the 1,203 sand flies captured. We found no flagellates in dissected females, but two pools containing females of *Ny. intermedia* presented DNA of *L. infantum*.

**CONCLUSION:**

Our results suggest that *Ny. intermedia* might be involved in the establishment of this new VL focus in Sao Paulo. However, before incriminating this species as a *L. infantum* vector, further studies should investigate other vectorial capacity parameters, including competence, survival, and feeding habits.

## INTRODUCTION

In America, Visceral Leishmaniasis (VL) is a neglected tropical disease (NTD) caused by the protozoan *Leishmania* (*Leishmania*) *infantum*, widely distributed from southern United States to Argentina^1,2^. The disease has been spreading in Brazil, especially in urban ecosystems due to the urbanization of *Lutzomyia longipalpis* species complex, its main vector ^3,4^. Such complex can find several blood feeding sources in the urban environment, including domestic dogs (*Canis familiaris*) – the main urban reservoir of *L. infantum*^5^. As a result of this agent-vector-host interaction, the incidence of both canine and human VL has increased in all the geographical regions of the country^6^, which recorded 27,233 cases from 2011 to 2018, representing an average of 3,404 new cases per year^10^.

Many factors have been associated with VL occurrence, including socioeconomic determinants such as poverty and limited access to health services^11^. However, factors related to the vector-host ecology and the landscape also help defining different transmission scenarios^3,14^. Two populations of the *Lu. longipalpis* complex have been reported in the state of São Paulo, namely Cembrene 1 and (S)-9-methylgermacrene-B^3^. Since the end of the 1990s, canine and human VL cases have been reported in areas where the (S)-9-methyllermacrene-B population is present^17^. Studies have shown that proximity to the Bolivia-Brazil gas pipeline and high annual average temperatures influenced *Lu. longipalpis* complex spread^18^. Moreover, the notification of infected dogs and their dispersion were associated with highways and previous reports of competent vectors^20^.

The dispersion of infected dogs is a key factor for the emergence of new foci of VL in locations with sand fly species, be them vectors of the LV agent or not^13^. Unlike areas where (S)-9-methylgermacrene-B and Cembrene 1 populations of *Lu. longipalpis* occur in São Paulo^3,21^, human VL cases were reported in the metropolitan region of São Paulo (Diadema municipality) in 1979^22^, and canine cases have been reported in the municipalities of Cotia and Embu das Artes since 2003 ^23^ – areas without the presence of *Lu. longipalpis* complex and where *Pintomyia fischeri* was the predominant sand fly species. Studies in these two municipalities have indicated *Pi. fischeri* as a potential vector of *L. infantum*^24^.

A recent focus of VL has been reported in the municipality of Guarujá, with three human cases and two deaths registered between 2016 and 2017^25^, without the presence of *Lutzomyia longipalpis*. Thus, this study sought identify the phlebotomine fauna and investigate natural infection by *L. infantum* in this focus sand fly species, contributing to VL surveillance.

## METHODS

### Study Area

**Figure 1:**
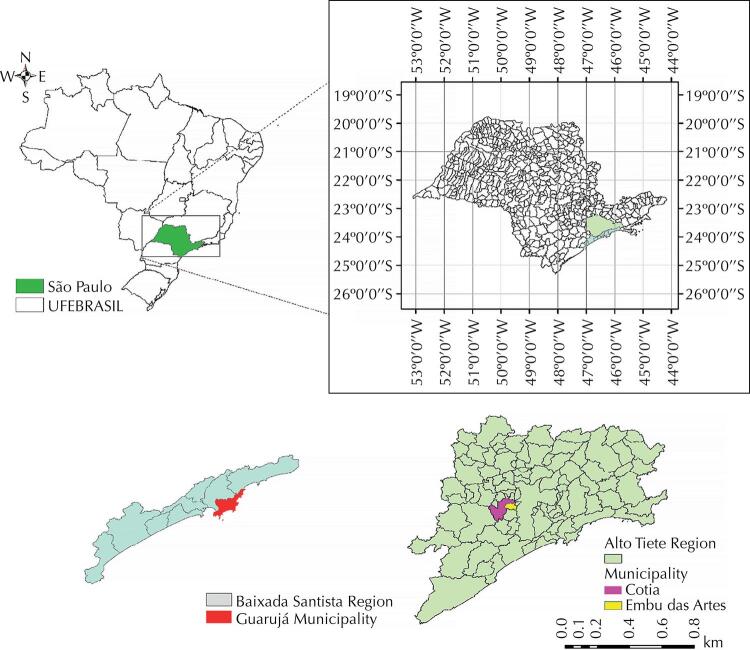
Geographical localization of São Paulo state, the Guarujá municipality, and the municipalities with canine VL cases reported in the Alto Tiete Region.

Guarujá (23º 59’ 35” S, 46º 15’ 23” W) is located on the coastal region of São Paulo (Fig. 1), with a mean altitude of 140 meters above sea level (MASL) and highest altitude of 300 m^26^. The municipality’s population is 380,000 inhabitants^27^, and its climate is classified as tropical wet Af according to Köeppen’s climate classification^28^, with an average annual temperature of 21.8 ºC and an average annual rainfall of 2,556 mm. The first autochthonous VL cases in this municipality were reported in 2016/2017, in the Balneário Cidade Atlântica, on Enseada beach, Morro da Enseada (23°58’02.0”S, 46°13’27.8”W), two of which affected children and another one a dog. This locality is situated in a high altitude area, whose slopes are covered with original Atlantic forest vegetation^26^. The population consists of low-income residents living in shacks built on the edge of the native forest, with shelters for domestic animals such as dogs and chickens – a result of the haphazard urban growth.

### Sand Flies: Collection, Identification, and Investigation of Flagellates and Leishmania infantum DNA

Collections were conducted from 5 pm to 8 am during 14 months, from November 2016 to September 2018, using light traps of the Center for Diseases Control (CDC). Traps were installed in the peridomicile of the house where cases had occurred, as well as in four nearby houses (about 300 meters away). Sand flies were transported to the LESP/Phlebotominae Laboratory of the School of Public Health of the University of São Paulo, where specimens were processed. For identification, males were clarified and mounted on slides, according to the method proposed by Forattini^29^, and females were dissected, as described in Diniz et al.^30^. Specimens were identified based on spermathecae and head characteristics during examination using a Galati’s key^31^.

Flagellates presence was investigated by dissecting some of the females captured and observing their guts under the optical microscope (400 X). To investigate the presence of *Leishmania* DNA, samples of these and other females that did not have their guts examined were grouped into pools, each consisting of a maximum of 20 females of the same species and that were collected in the same place and day. While awaiting the DNA extraction process, specimens were stored in 1.5 mL microtubes with isopropyl alcohol at 4 °C. To calculate the frequency of sand flies by month of capture, the numbers of males and females were plotted on an Excel matrix. Average monthly rainfall was obtained from the Accuweather website^32^.

### Sand Fly DNA Extraction

DNA extraction was performed for individual samples as well as for pools of up to 30 sand fly specimens using a QIAamp DNA Mini Kit (Qiagen) according to the manufacturer’s instructions, as described by dos Santos Brighente et al.^33^. DNA concentration and purity were assessed by the ratio of optical density (OD) at 260 and 280 nm using the NanoDrop ND1000 (Thermo Scientific). DNA was stored at -20 °C until analysis.

### PCR for Detection of *Leishmania* DNA

*Leishmania* genus and *L*. (*L*.) *infantum* were identified by polymerase chain reaction (PCR), using the following primers: 150- 5’GGGKAGGGGCGTTCTSCGAA3’ and 152-5’SSSWCTATWTTACACCAACCCC3’ for *Leishmania* genus^34^ and RV1- 5’CTTTTCTGGTCCCGCGGGTAGG3’ and RV2 -5’CCACCTGGCCTATTTTACACCA3’^35,36^ for *L*. (*L*.) *infantum*. While the first primer amplifies the kDNA minicircle conserved region of *Leishmania* sp., the last one amplifies the kDNA minicircle variable region of *L. donovani* complex. PCR were performed with 25 μL Go Taq Green Master Mix (Promega) containing 1μM of each primer and 3 μL of DNA. All reactions included one positive (DNA extracted from L. infantum, reference strain MHOM/BR/74/PP75) and one negative control (ultrapure water). To assure the quality of the extracted DNA and the integrity of negative results, all samples were tested using the primers Lu.18S 1S- TGCCAGTAGTTATATGCTTG and Lu.18S AR- CACCTACGGAAACCTTGTTAC, which amplify the 18SrRNA gene – a conserved region of *Lutzomyia* species^37^. In these tests, PCR were performed in a final volume of 25 μL containing 0,45μM of each primer and 1 μL of DNA. PCR products were electrophoresed in 2% agarose gel stained with ethidium bromide and visualized under UV illumination.

## RESULTS

*Ny. intermedia* was the most prevalent species (n = 1,091) within the 1,203 collected specimens, representing 90.7% of the total. The least common species among the study sample were *Migonemyia migonei* (5.2%), *Psathyromyia pascalei* (3.1%), *Pintomyia fischeri* (0.9%), and *Psychodopygus ayrozai* (0.1%).

**Figure 2:**
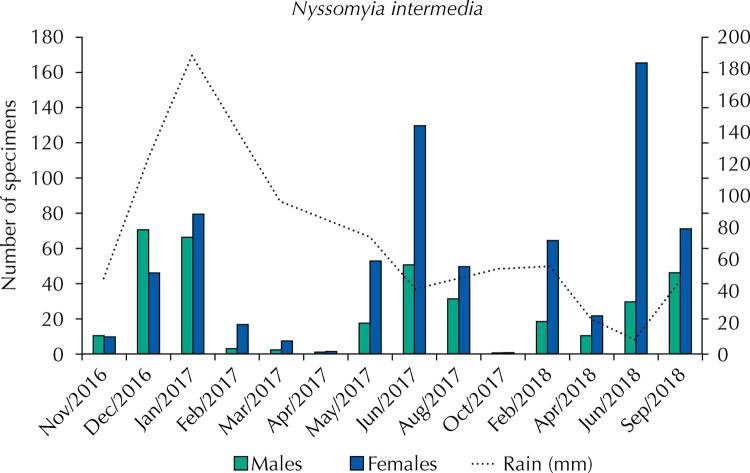
Monthly distribution of rainfall and number of *Nyssomia intermedia* sand flies collected from November 2016 to September 2018 in Morro da Enseada, Balneário Cidade Atlântica, Enseada beach, Guarujá, São Paulo, Brazil.

*Nyssomyia intermedia* was the only species present in all captures, so that this study description of sand flies monthly distribution refers to that species. In total, 723 females (66.3%) and 368 males (33.7%) of *Ny. intermedia* were captured throughout the study period (Table 1), with higher frequencies in the dry season, during June 2016 (16.6%) and June 2017 (18.0%), which were also the months with the lowest rainfall averages (Fig. 2).

Samples of all species captured were analyzed by dissection and/or PCR assay. Regarding *Ny. intermedia,* 332 females (46%) were dissected and had their guts examined for the presence of flagellates. The same was performed with females of other species, including *Mg. migonei* (19/28), *Pa. pascalei* (3/26), *Pi. fischeri* (2/11), and *Ps. ayrozai* (1/1). However, we found no flagellates in dissected specimens.

In total, 345 females of all species underwent PCR analysis to detect the presence of *Leishmania* spp and *L. infantum* DNA. These females were captured in January and June 2017; and February, April, June, and September 2018 – an interval that includes both rainy and dry seasons. We analyzed 28 pools containing 337 females of *Ny. intermedia* (Table 1), as well as three pools and individual samples (n = 4) of *Mg. migonei*, and individual samples of *Pi. fischeri* (n = 2), *Ps. ayrozai* (n = 1), and *Pa. pascalei* (n = 1).

*Lutzomyia* spp. housekeeping gene was amplified in all DNA samples.

We verified the presence of *Leishmania* DNA in two pools of *Ny. intermedia*: one of them containing 17 specimens (minimum rate: 1/17; 6.0%) collected in January 2017 and the other containing 20 specimens (minimum rate 1/20; 5.0%) collected in June 2018. Using the specific primers RV1/RV2 to detect *L. donovani* complex DNA, we verified the occurrence of *L. infantum* in both pools. Thus, the minimum rate of females of *Ny. intermedia* carrying *L. infantum* DNA was 1.9% among the total number of females examined in January 2017 (n = 42); 1% among females examined in June 2018 (n = 100); and 0.59% among the total sample (2/337). We detected no *Leishmania* DNA in the samples of *Mg. migonei, Pi. fischeri, Pa. pascalei,* and *Ps. ayrozai*. A total of 187 (55.5%) of females examined by PCR were also dissected, with negative results for both flagellates and *L. infantum* DNA.

## DISCUSSION

When investigating specimens captured from a new focus of VL in Guarujá, where human and canine cases have been reported, we verified the predominance of *Ny. intermedia* sand flies and the presence of *L. infantum* DNA^38^. We captured no specimens of *Lu. longipalpis* during the study period, indicating that VL transmission in this area occurs by means of other sand fly species. Considering that density influences vector capacity^15,39^ and that *Ny. intermedia* was the only species present in all captures, besides always being the most numerous, we may rightfully infer that this species participates in *L. infantum* transmission within this focus. Other potential vectors of *L. infantum* were identified in the area, such as *Pi. fischeri* and *Mg. migonei*^24,40^; however, these species were low in density, indicating that they could not possibly maintain the transmission cycle – only 5% of females captured were of *Mg. migonei*, and the number falls even further with *Pi. fischeri*, accounting for 0.9%.

*Nyssomyia intermedia* is a widespread sand fly species in Brazil, especially in the Atlantic Forest biome (Galati, 2018). As reported by Brito et al.^41^ in a study conducted in a northern coastal area of São Paulo, its highest frequencies occur in the dry season (Figure 2). However, *Ny. intermedia* occurrence peaked in both dry and humid seasons in the Ribeira Valley, a transitional area between the Atlantic plateau and the coastal plain in southeastern São Paulo, without significant correlation with the rainfall registered in the 30 days before each collection^42^ The same occurred for a savannah area in Minas Gerais, where no clear seasonal distribution was observed for this species^43^. Such differences may possibly account for the degree of proximity to the Atlantic Ocean, vegetation cover, and observation interval.

Although recognized as a vector of *Leishmania braziliensis*^1,44^, a previous study conducted with a focus of VL in Minas Gerais detected *L. infantum* DNA in *Ny. intermedia* (1/7; 14.3%), where *Lu. longipalpis* was found harboring *L. infantum* flagellates^43^. In our study, we found *L. infantum* DNA in two pools of *Ny. intermedia*, representing a minimum infection rate of 0.6% when considering the overall collection period and of 1.4% when considering females examined in the two months with positive results. These results suggest that *Ny. intermedia* could be a permissive vector^45,46^, for it might be enabling the development of two different *Leishmania* species. Despite the high sensitivity of molecular methods in investigating the presence of *Leishmania* DNA, promastigotes are not necessarily alive^47^ nor will they survive the extrinsic incubation period. In this case, dissections enable the identification of flagellates and their development in metacyclogenesis. In our study, we found no flagellates in the gut of dissected females (55.5% of the females captured). Considering that some species can develop early *Leishmania* stages until blood digestion, when parasite development may fail^46^, and that *Leishmania* DNA detected through PCR may be due to blood meals ingested by sand flies^48^, further studies are required to evaluate these aspects.

Our results highlight the need for further information on the distribution of *L. infantum* permissive species, thus enabling a more precise identification of risk areas and more effective control programs. Although the detection of *L. infantum* DNA in predominant species and the lack of *Lu. longipalpis* do not suffice to incriminate this focus vector, our findings introduce elements that reinforce the suspicion as to the role of *Ny. intermedia* as a vector of this parasite. Regardless, further studies should evaluate other parameters of its vector capacity, including its competence. VL surveillance and control strategies recommended by the Brazilian Ministry of Health are directed to transmission areas with the presence of the *Lu. longipalpis* complex and *Lu. cruzi*. However, before the possibility of introduction of infected dogs, which may lead to the establishment of new transmission cycles, our results stress the need for expanding entomological surveillance to areas where other potential vectors of *L. infantum* occur.

We found *Ny. intermedia* species to play a potential role in the transmission of *L. infantum* within the focus of LV in Guarujá, reinforcing the need for entomological surveillance in locations where this sand fly species occurs to support the decisions made by the health authorities involved in the VL control program. Before incriminating this species as a *L. infantum* vector, further studies should investigate its vector capacity parameters, including competence, survival, and feeding habits.

**Table:** Distribution of specimens of *Nyssomyia intermedia* according to sampling month and number of females captured in Guarujá-SP from November 2016 to September 2018 analyzed by dissection or PCR.

Collection date	Female n	Male n	Total	Dissected	PCR Examined	Examined by dissection and PCR (%)
November/2016	10	11	21	0	0	1.4
December/2016	47	71	118	0	0	6.5
January/2017	80	67	147	30	42	11.1
February/2017	17	4	21	0	0	2.4
March/2017	8	3	11	8	8	1.1
April/2017	2	2	4	0	0	0.3
May/2017	53	18	71	0	0	7.3
June/2017	130	51	181	71	29	18.0
July/2017	50	32	82	0	0	6.9
October/2017	1	1	2	0	0	0.1
February/2018	65	19	84	65	65	9.0
April/2018	22	12	34	22	21	3.0
June/2018	166	30	196	66	100	23.0
September/2018	72	47	119	70	66	10.0
Total	723	368	1,091	332	331	100
